# Another Screw Loose

**Published:** 1860-02

**Authors:** 


### ANOTHER SCREW LOOSE.

In the January number of the *American Journal*, we have another Jeremiad from the pen of him who recently sang a tearless requiem over the departed glory of our " Dental Periodical Literature." Now he is disconsolate over the failure of dental societies. And here he can not even comfort himself by a reference to the past, as he did in regard to dental periodicals; for dental societies, he maintains, have always "been marked by failure." So decided does he consider the failure, that he is almost ready to call the whole movement a " contemptible effort."

This writer proposes, in the outset, to survey " the work done by those National Dental Societies which have from time to time, been ushered into an ephemeral existence;" and, had he confined himself to this, we would have rested content. But when he calls the work done by the two national associations a *failure*, or a *contemptible effort*, and at the same time claims they have done all that has been accomplished, he does injustice to other societies. Of the local societies he says—" but of true scientific fruit they produced nothing. The parent tree bore all that was to be gathered." The parent tree, here mentioned, is the same national society whose course from the beginning "has been marked by failure;" but now we are told, not that it bore all the fruit that *was* gathered, but "all that *was to be gathered.*" Is a tree that monopolizes all the fruit of the orchard, bears all that is to be gathered, a failure?

But have the local societies produced no fruit? The writer, under consideration has "been able to discover two circumstances which reflect credit upon the national societies; and the prominent one of these "relates to the pecuniary assistance extended to a periodical." But, by "rigid search," he would have been reminded that one of the local societies started and sustained a periodical till it became self-sustaining. Every dentist knows that the "Mississippi Valley Association" is the parent of the *Dental Register.* But the same society bore other fruits. Among these may be enumerated cylinder or block fillings, continuous gum work, the swaged socket rim in plate work, the warm air blow-pipe, the best, and generally adopted method of using crystal gold, the most simple and convenient non-explosive spirit-lamp, the improved sherwood forceps (which leave every thing else out of sight behind); Talbert's drill excavators, and the use of definite retaining points, (now in general use,) *adhesive foil* (by Dr. Leslie), the separable screw for extracting fangs (more convenient and better than the screw forceps), the *rational* use of creosote, chloride of zinc, nitrate of silver, and other agents applied to the teeth, a popular essay, which has gone through four editions, besides hosts of other things which we could mention by referring to documents. These we have penned as we thought of them, without any effort at arrangement or classification of them.

Another local society erected the first building, and the only one the world ever saw expressly built for the purpose of dental instruction; and no building in the world is better adapted to the purpose for which it was designed. And much might be said of the honorable and useful efforts of other local societies; but we must refrain. The above will show how little credit is to be given to such sweeping assertions as this writer seems fond of.

It is not uncommon for men to become misanthropic and morose; and when they do so they conclude that all is wrong. Examples are numerous in the church, in the state and in the profession. If the disaffected member of our profession is an old man, he gravely concludes and mournfully maintains that the profession is going backward in manners, in morals and in science, when perhaps the whole difficulty is that he has lost his way, has been disappointed, and, in his confusion, has taken the back track himself, and seeing no others going the same way, concludes that all have forsaken the cause; and proved recreant to their trust. If he is a young man, he concludes that all who do not believe his vague theories and respect his whims, are false to the profession and hostile to himself. He imagines that he is almost a martyr, that he is persecuted for opinion's sake, that whenever he speaks others are for war, that they even wish to deprive him of that lawful inheritance, the correct use of his mother tongue; and he sincerely believes that the only hope of the profession lies deep down in the recesses of his mind. Accordingly, he rebukes young men for their opinions, tells old men *his experience*, and admonishes all that it is a very serious thing to differ with him in judgment.

Such we believe to be a fair description of the old croaker, and the young croaker. We have no sympathy with croakers in any department of society. They are all impracticable and useless. The pious croaker laments that religion is perishing from the earth, though God's faithfulness is pledged to sustain it. The political croaker cries "the Union is in danger," though it is ten times stronger than when consumated. The dental croaker laments that our profession is doing nothing —is even retrograding, while its progress eclipses that of any other profession in the world. And the writer of the article under consideration mourns over the failure of dental societies, and regards their proceedings as "*lamentably* invaluable,' while, in truth, they have done more to accomplish the ends for which they were organized than any other associations of the same period. The jaundiced man concludes that every thing is yellow but himself; and the drunken man thinks that even the lamp post is staggering from the effects of strong drink.

Even the arithmetic of our friend in the *Journal*, seems to be subjected to the same kind of perversion, giving evidence that he labors to some extent under a mathematical halucination. For example, in speaking of the number of essays read at the early meetings of the "American Society," he says: "The gradual diminution in the number appointed and *read continued.*" Now let us examine this gradual *diminution*, which preceded the meeting he refers to. At the first meeting after the organization, according to his showing, "not one was read." By the next meeting they had *gradually diminished up to three*, and by the next, to "three or *four.*" This is a small affair, but we mention it to show how ludicrous men make themselves, when determined to complain, while they have nothing to complain of.

Of the two national societies this writer tells us that "their sole result has been to show that the right course is directly opposite to the one heretofore followed." But it is plain that the two societies under consideration followed different courses—as nearly opposite as could well be conceived. To which one of these is the right course directly opposite? Or perhaps *direct* opposition would preclude all association whatever. If so, a great many are following the right course.

But the old society having reached "its full vigor," we are told that it "began rapidly to decline," and that it "tottered through two or three years of *puerility* and died." Reached its full vigor and failed into *boyhood.* How many would be happy to fail in the same direction!

To sum up the views of the writer in regard to the various meetings of the "American Convention," we might say that he considers the first "unhealthy," the second entitled to "all due praise," the third "a feeble imitation" of the second, the fourth "without any feature of interest whatever," and the fifth, as "noticeable for the good opinion some of the members appeared to entertain of it.

Why the second meeting was so good, and the fourth "without any feature of interest," the reader may be at a loss to determine. It is probable that the writer of the article under consideration was at the second meeting but was absent from the fourth. Had he been present, we think he would have succeeded in making the fourth almost as interesting as the second.

But for all the evils of past and present associations, the writer suggests a remedy. Assuming that the great evil has been "freedom of access," he directs his remedy against this. He tells us that "it is certain that our chief evil may be greatly mitigated by the money test. There can be no doubt that considerable fees will keep out a large class of persons who are not of the least use, and who, under the present order of things, are exceedingly troublesome."

This money test might do pretty well in practice, if the unworthy were too poor or too penurious to pay "considerable fees," and if, on the other hand, the worthy were all able and willing to comply with such exactions. But the writer himself admits that his "money test" will not reach a "much to be dreaded" class, who "are willing to make large outlays in the gratification of vanity, who *must* be known as participants in every movement." "The five or ten minutes rule is the only check" he can think of to meet their case. That is, to keep the fools who *buy* their way in from *boring* him, he will allow the wise to say little or nothing. Enough said.

